# Microsatellite marker analysis of *Haemonchus contortus* populations from Pakistan suggests that frequent benzimidazole drug treatment does not result in a reduction of overall genetic diversity

**DOI:** 10.1186/s13071-016-1624-0

**Published:** 2016-06-17

**Authors:** Umer Chaudhry, E. M. Redman, Kamran Ashraf, Muhammad Zubair Shabbir, Muhammad Imran Rashid, Shoaib Ashraf, John S. Gilleard

**Affiliations:** Department of Comparative Biology and Experimental Medicine, Faculty of Veterinary Medicine, University of Calgary Alberta, Calgary, Canada; Department of Parasitology, University of Veterinary and Animal sciences Lahore, Punjab, Pakistan; Quality Operations Laboratory, University of Veterinary and Animal Sciences, Lahore, Pakistan; Department of Pharmacology, University of Veterinary and Animal sciences Lahore, Punjab, Pakistan; Present Address: Roslin Institute, Royal (Dick) School of Veterinary Studies, University of Edinburgh, Scotland, UK

**Keywords:** *Haemonchus contortus*, Helminth, β-tubulin, Anthelmintic, Drug resistance, Benzimidazole, Albendazole, Soft selective sweep, Genetic diversity

## Abstract

**Background:**

The impact of drug selection pressure on the overall genetic diversity of parasitic nematode populations in the field is poorly understood. In this study, we address this issue for the small ruminant parasite *Haemonchus contortus* in the Punjab, Pakistan. This region provides an opportunity to compare *H. contortus* populations that have been subjected to a prolonged period of frequent benzimidazole drug treatments on government farms with parasite populations that have been exposed to little or no drug treatment in neighbouring pastoral herds.

**Methods:**

Adult *H. contortus* worms were collected from the abomasa of small ruminants from three government farms frequently using benzimidazole drugs, and closed to animal movement, for over 30 years and also from from eighteen pastoral herds subject to minimal drug selection. The frequency of three known benzimidazole resistance associated mutations was determined in each parasite population. For the seven parasite populations in which resistance mutations were found, the diversity, geographical distribution and phylogenetic relationships of isotype-1 β-tubulin benzimidazole resistance haplotypes were determined. In addition, the genetic diversity of the parasite populations on the three government farms were compared with those from four pastoral herds.

**Results:**

The F200Y (T**A**C) resistance mutation was present at a very high frequency in *H. contortus* populations from government herds, but not from pastoral herds, consistent with their respective drug selection histories. Population genetic analysis, using a panel of microsatellite markers, revealed that there was little genetic differentiation among the parasite populations with no significant difference in the overall genetic diversity between government and pastoral herds. In addition, sequence analysis of the isotype-1 β-tubulin locus revealed multiple F200Y (T**A**C) haplotypes demonstrating soft selective sweeps even in government herds with little or no contemporary parasite migration.

**Conclusions:**

The results suggest that, although the frequent drug treatment used on government farms has selected for a high frequency of benzimidazole resistance mutations, there has been little or no reduction in the overall genetic diversity of the selected parasite populations.

**Electronic supplementary material:**

The online version of this article (doi:10.1186/s13071-016-1624-0) contains supplementary material, which is available to authorized users.

## Background

Anthelmintic resistance in parasitic nematodes is a global threat to sustainable livestock production [1]. However, we still have a limited understanding of how drug selection affects parasite populations and how resistance emerges at the population level. *Haemonchus contortus* is one of the most economically important helminth parasites of small ruminants worldwide and is an important model for anthelmintic resistance research [2]. This parasite has developed resistance to all the major anthelmintic drug classes and resistance to multiple drug classes occurs, often at high frequency, in many parts of the world [3, 4].

Benzimidazole drugs have been used to control livestock parasites for over 40 years and resistance to this drug class is at an advanced stage in many parts of the world [5, 6]. We have sufficient knowledge of the molecular basis of resistance for this drug class in order to study the emergence and spread of anthelmintic resistance mutations in field populations. Three single nucleotide polymorphisms (SNPs) in the isotype-1 β-tubulin gene have been associated with benzimidazole resistance in *H. contortus*. The F200Y (T**T**C to T**A**C) mutation has been found in every country examined to date and is often present at high frequency [5–9]. We have recently presented genetic evidence that this mutation independently arises multiple times in a geographical region and spreads between sites by migration [5, 10]. The SNPs at codons F167Y (T**T**C to T**A**C) and E198A (G**A**A to G**C**A) have also been reported in multiple countries but have a more variable occurrence and are generally present at lower frequency than the F200Y (T**T**C to T**A**C) mutation [5–7, 11]. We have recently provided genetic evidence to show that E198A (G**C**A) mutation present in multiple populations in southern India was derived from a single origin [10].

We are investigating the population genetics of benzimidazole resistance in *H. contortus* in a variety of different countries to provide insights into its origin and spread [5, 6, 10, 12]. Benzimidazole resistance is at an advanced stage in most countries that we, and others, have examined to date due to the widespread use of this drug class over many years. In places such as the United Kingdom (UK), western Europe, Australia and North America most *H. contortus* populations have moderate to high levels of benzimidazole resistance [5–7]. In these countries, most parasite populations have at least one of the resistance-associated mutations at high frequency. We have been seeking locations where we can investigate benzimidazole resistance at an earlier stage of its emergence and Pakistan is a potentially interesting country in this respect. Economic constraints mean that most farmers do not use specific anthelmintic treatment regimes, anthelmintics are often diluted before administration and generic drugs of unknown quality are often used. Consequently, we anticipated that benzimidazole resistance might be at a relatively early stage of emergence in animals from small rural farms or owned for subsistence purposes. In contrast, government farms tend to use frequent treatments with benzimidazole drugs providing a potentially interesting contrast in the same geographical region. There are only a few published studies on benzimidazole resistance in Pakistan but there is information to suggest resistance may not yet be widespread [13, 14]. Fecal egg count reduction tests performed on Beetal goats on eighteen farms in the Faisalabad district of the Punjab showed oxfendazole efficacies of between 80–100 % (over 95 % fecal egg count reduction on eleven on the farms) [13]. The sequencing of an isotype-1 β-tubulin gene fragment from 95 adult *H.contortus* worms collected from abattoirs in the Punjab and North West Frontier provinces revealed just a 7.86 % frequency of the F200Y (T**A**C) mutation [14].

In the present paper, we show that the F200Y (T**A**C) benzimidazole resistance mutation is present at very low frequencies in eighteen *H. contortus* populations sourced from sheep and goats from rural locations in the Punjab Province of Pakistan. In contrast, this mutation is present at very high frequencies in three government farms that have used frequent and regular anthelmintic treatments for over 30 years. In spite of this difference in drug selection, population genetic analysis, using a panel of microsatellite markers, revealed no significant difference in the overall genetic diversity between the government and pastoral herds. In addition, phylogenetic analysis of isotype-1 β-tubulin haplotypes reveals multiple F200Y(T**A**C) haplotypes are present in *H. contortus* on the closed government herds suggesting the presence of soft selective sweeps in the absence of significant contemporary parasite migration.

## Methods

### Parasite collection

**Fig. 1:**
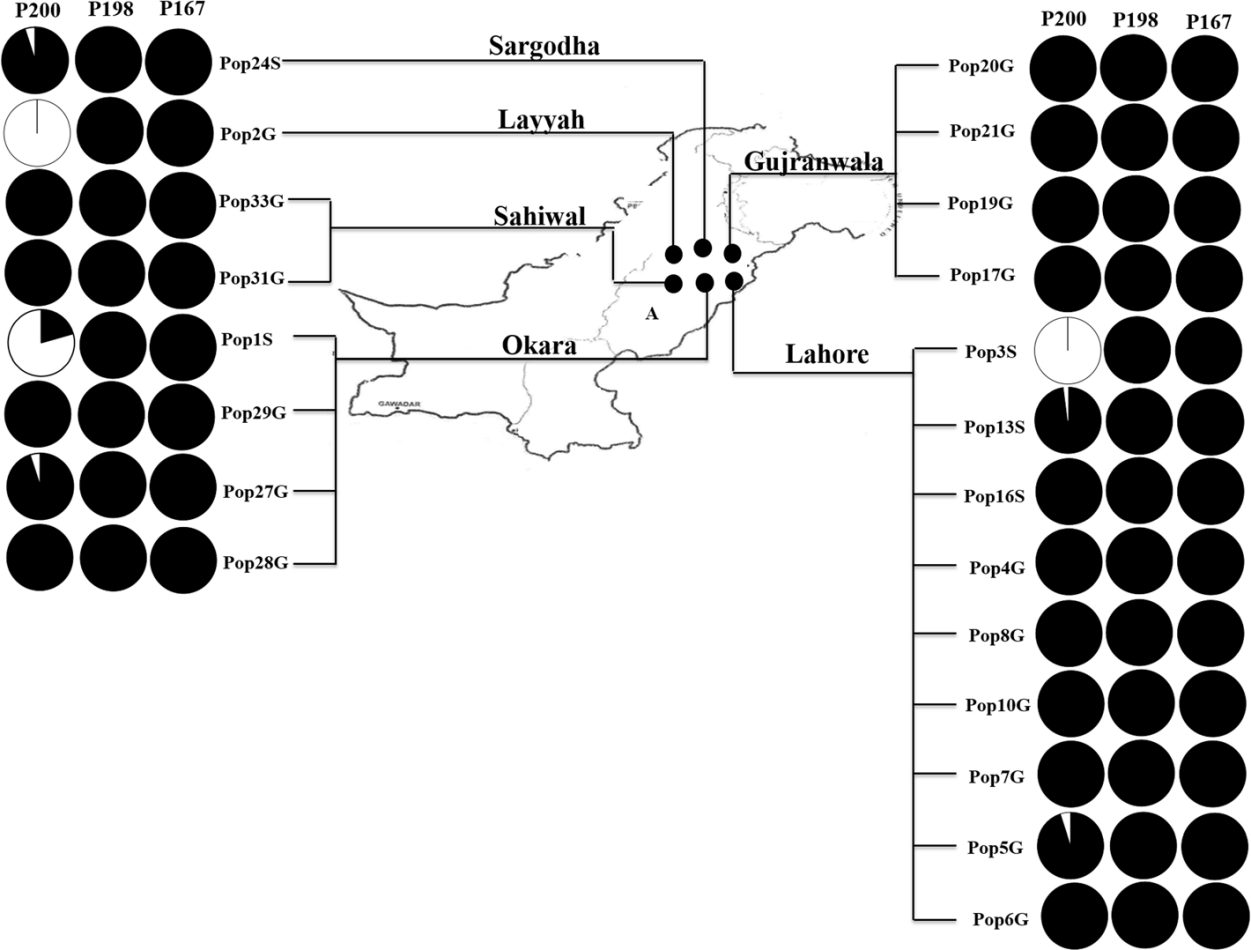
Relative frequencies of the F200Y (T**A**C), F167Y (T**A**C) and E198A (G**C**A) isotype-1 β-tubulin benzimidazole resistance mutations in *H. contortus* populations from the Punjab region of Pakistan. Each *H. contortus* population is represented by three pie charts; one for each resistance mutation; F200Y (T**T**C/T**A**C), F167Y (T**T**C/T**A**C) and E198A (G**A**A/G**C**A). Each of these pie charts shows the relative frequency of the resistant versus susceptible SNP, based on allele quantification by pyrosequence genotyping of pooled DNA from between 14–32 worms per population (Additional file 1: Table S1). The resistance-associated SNP genotype frequency - F200Y (T**A**C) - is shown in white and the “susceptible” SNP genotype frequencies - F200Y (T**T**C), F167Y (T**T**C) and E198A (G**A**A) - are shown in black. Geographic locations of abattoirs are indicated with small circles on the map and the abattoir names from which the samples were obtained are shown above the labelling lines. The province is indicated on the map (A) Punjab

Adult *H. contortus* worms were harvested from the abomasa of eighteen individual ruminant hosts, known to originate from different pastures/villages in the Punjab province of Pakistan in 2012, from abattoirs who source local animals (Fig. 1; Additional file 1: Table S1). Adult worms, were also obtained from necropsied animals from three government farms in the Punjab. Five of these *H. contortus* populations were collected from sheep comprising two from the Lahore abattoir (Pop16S and Pop13S), one from the Sargodha abattoir (Pop24S), one from the Jahangirabad government farm (Pop3S) and one from the Okara government farm (Pop1S). Sixteen of these *H. contortus* populations were collected from goats comprising six from the Lahore abattoir (Pop5G, Pop7G, Pop6G, Pop4G, Pop8G, Pop10G), three from the Okara abattoir (Pop27G, Pop28G, Pop29G), two from the Sahiwal abattoir (Pop31G, Pop33G), four from the Gujranwala abattoir (Pop17G, Pop19G, Pop20G, Pop21G) and one from the Layyah government farm (Pop2G) (Fig. 1: Additional file 1: Table S1).

The Layyah government farm (pop2G) was established in 1980 from angora goats sourced from Turkey which were crossed with locally sourced goats of the “hairy breed”. This herd has been treated with anthelmintics (levamisole HCL and oxfendazole) approximately every 3 months since its establishment and has been closed to animal movement since that time. The Okara (Pop1S) and Jahangirabad (Pop3S) government farms were established using local sheep breeds (lohi and kajli) in 1985 and 1989 respectively. Both these herds have been treated alternately with albendazole and oxfendazole approximately every 3 months since their establishment. There has been some historical movement of sheep between the Okara and Jahangirabad farms but these herds have otherwise been closed to animal movement since they were established.

### Genomic DNA isolation and species confirmation

Adult worms were fixed in 70 % ethanol immediately following removal from the host abomasum. The heads of individual worms were dissected and lysed in single 0.2 μl tubes containing 50 μl of proteinase K lysis buffer and stored at -80 °C as previously described [5, 15]. One microliter of 1:5 dilution of neat single worm lysate was used as PCR template and identical dilutions of lysate buffer, made in parallel, were used as negative controls. To prepare pooled lysates of each population, 1 μl aliquots of each individual neat adult worm head lysate were pooled. One microliter of a 1:20 dilution of pooled lysate was used as template for subsequent PCRs.

A combination of gross morphological identification and genotyping of the SNP at position 24 of the ITS-2 rDNA was used to confirm the identity of *H. contortus* in these populations as previously reported [15]. A minimum of 14 and a maximum of 32 individual adult *H. contortus* worms were identified per population (560 *H. contortus* worms in total). Individual *H. contortus* DNA lysates were pooled for each population for isotype-1 β-tubulin genotyping.

### Pyrosequence genotyping to determine the relative frequencies of the isotype-1 β-tubulin F200Y(TAC), E198A(G**C**A) and F167Y(T**A**C) benzimidazole resistance-associated SNPs in *H. contortus* populations

A 328 bp fragment, spanning exons 4 and 5 and intervening intron of the isotype-1 β-tubulin gene was PCR amplified from pooled DNA lysates for each of the 21 *H. contortus* populations (Additional file 1: Table S1). Final PCR conditions were 1X thermopol reaction buffer, 2 mM MgSO_4,_ 100 μM dNTPs, 0.1 μM forward and reverse primers and 1.25U Taq DNA polymerase (New England Biolabs, Ipswich,USA) and a previously published primer pair was used; forward primer (HcPYRF: 5′-GAC GCA TTC ACT TGG AGG AG-3′) and reverse primer (HcPYRR: 5′-Biotin-CAT AGG TTG GAT TTG TGA GTT-3′) [16]. The thermo-cycling parameters consisted of an initial 95 °C for 5 min followed by 35 cycles of 95 °C for 1 min, 53 °C for 1 min and 72 °C for 1 min with a single final extension cycle of 72 °C for 5 min.

Following PCR amplification, the relative frequency of the F167Y (T**A**C), E198A (G**C**A) and F200Y (T**A**C) isotype-1 β-tubulin SNPs in the amplicons derived from each parasite population were determined by pyrosequence genotyping using the PryoMark ID system using the allele quantification (AQ) mode (Biotage, Uppsala, Sweden). Previously published sequencing primers were used for the F167Y (T**A**C), E198A (G**C**A) and F200Y (T**A**C) mutations; Hcsq167: 5′-ATA GAA TTA TGG CTT CGT-3′, Hcsq198: 5′-ACT GGT AGA GAA CAC CG-3′ and Hcsq200: 5′- TAG AGA ACA CCG ATG AAA CAT-3′, respectively [8, 10, 16]. The base dispensation was set to GATGCTCGT for codon 167, GATGCAGCA for codon 198, and GATGCTGTA for codon 200. Peak heights were measured using the AQ mode of the PSQ 96 single nucleotide position software as previously described [10].

### Cloning and sequencing of an isotype-1 β-tubulin fragment encompassing the F200Y (T**A**C), E198A (G**C**A) and F167Y (T**A**C) mutations

The same 328 bp isotype-1 β-tubulin fragment described above was also amplified, cloned and sequenced from the pooled lysates from the seven *H. contortus* populations in which the F200Y (T**A**C) was detected (Pop24S, Pop1S, Pop27G, Pop2G, Pop3S, Pop5G and Pop13S). Primers HcPYRF: 5′-GAC GCA TTC ACT TGG AGG AG-3′ and HcPYRR: 5′-CAT AGG TTG GAT TTGTGA GTT-3′ were again used but with the following PCR reaction conditions; 5X Phusion HF reaction buffer, 2 mM MgSO_4,_ 200uM dNTPs, 0.2uM forward and reverse primers and 1U of Phusion high fidelity DNA polymerase (Finenzyme inc., Keilaranta, Finland). The thermo-cycling parameters of isotype-1 β-tubulin consisted of an initial 98 °C for 30 s followed by 35 cycles of 98 °C for 10 s, 59 °C for 30 s and 72 °C for 1 min with a single final extension cycle of 72 °C for 5 min. Amplicons were cloned into PJET 1.2/BLUNT vector (Thermo Scientific, Waltham, USA) and sequenced using previously described standard procedures [5]. Sequences were aligned with the *H. contortus* isotype-1 β-tubulin sequence (Acc. No. X67489) and edited using Geneious Pro 5.4 software [17]. A previously described approach was used to bioinformatically filter the isotype-1 β-tubulin sequences to remove SNPs occurring only once in the dataset and ensure PCR-induced mutations were not included in the analysis [5, 10].

### Phylogenetic network analysis of *H. contortus* isotype-1 β-tubulin sequences

Networks based on genetic distance were computed using the neighbour-net method employed in SplitsTree4 [18] to produce circular (equal angle) split networks. Median joining networks were generated in Network 4.6.1 software (Fluxus Technology Ltd., Clare, England). A full median network containing all possible shortest trees was generated by setting the epsilon parameter equal to the greatest weighted distance (epsilon = 10). All unnecessary median vectors and links were removed with the MP (Maximum Parsimony) option [19]. A phylogenetic network tree of the isotype-1 β-tubulin haplotypes was reconstructed using maximum likelihood (ML) in MEGA5 [20]. The program jModeltest 12.2.0 [21] was used to select the appropriate model of nucleotide substitutions for ML analysis. According to Bayesian information criterion the best scoring was Hasegawa-Kishino-Yano (HKY + G). The model of substitution was used with parameters estimated from data. Branch supports were obtained by 1,000 bootstraps of the data. The most probable ancestral node was determined by rooting the networks to a closely related outgroup and *Haemonchus placei* sequence was used to root the *H. contortus* network.

### Microsatellite genotyping and population genetic analysis

Previously described *H. contortus* microsatellites were screened to produce a panel of eight markers that consistently amplified from the Pakistan populations under study (data not shown) [5, 22, 23]. A summary of primer sequences and allele ranges are given in Additional file 2: Table S2 and the PCR conditions for each marker were as previously described [22]. The forward primer of each microsatellite primer pair was 5′ end labelled with fluorescent dye (IDT, Canada) and the GeneScan ROX 400 internal size standard was used on the ABI Prism 3100 genetic analyser (Applied Biosystems, Carlesbad, California, USA). Individual chromatograms were analysed using Gene Mapper software version 4.0 to accurately size the amplicons and determine genotypes (Applied Biosystems, USA).

Fixation index (pairwise F_ST_) values were calculated from the multi-locus microsatellite genotype data, by random permutation in Arlequin 3.11 [24]. Exact test was used to statistically evaluate deviations from Hardy-Weinberg equilibrium for all populations [25]. Significance levels were adjusted using the sequential method of Bonferroni for multiple comparisons in the same dataset [26]. There was a significant departure from Hardy-Weinberg equilibrium, even after Bonferroni correction, for 10 out of the 56 loci combinations for *H. contortus* (Additional file 3: Table S3). The presence of null alleles for microsatellite loci has been previously reported and is the likely reason for these departures from Hardy-Weinberg equilibrium [22, 27–29]. Analysis of molecular variance (AMOVA) was estimated through partition of microsatellite diversity between and within populations [30]. Log-likelihood ratio test statistic (G-test), used to estimate the linkage disequilibrium analysis, was carried out by Arlequin 3.11 [24]. Expected and observed heterozygosity (H_e_ and H_o_), allele richness and estimation of inbreeding coefficient (F_IS_) for each locus were calculated using Arlequin 3.1 (Additional file 3: Table S3) [24]. As the number of alleles and expected heterozygosity are dependent on sample size, in order to compare populations, expected heterozygosity (H_expc_) and allele richness (A_c_) were also calculated per locus for each population with correction for unequal sample size using POPTREE2 [31] and MSA 4.05 [32], respectively. Differences in these measures of genetic diversity between populations were tested for statistical significance using a Wilcoxon rank sum test [33].

## Results

### The F200Y (T**A**C) isotype-1 β-tubulin mutation was found at high frequency in *H. contortus* populations on government farms but only rarely from animals from pastoral locations

A 328 bp fragment of the isotype-1 β-tubulin gene was PCR amplified from 21 *H. contortus* populations (3 populations from government farms and 18 populations from pastoral locations) and the relative frequencies of the three currently known benzimidazole resistance-associated SNPs F167Y (T**A**C), E198A (G**C**A) and F200Y (T**A**C) was determined by pyrosequence genotyping (using pools of 14 to 32 worms per population; see Additional file 1: Table S1). The benzimidazole resistance-associated F200Y (T**A**C) SNP was found at high frequency (81–100 %) in all three parasite populations from government farms. In contrast, it was not detected in 14 out of the 18 pastoral populations and only detected at low frequency (2–6 %) in the remaining four (Fig. 1 and Additional file 1: Table S1). The F167Y (T**A**C) and E198A (G**C**A) SNPs were not detected in any of the populations.

### Genetic diversity and population structure of *H. contortus* populations

**Fig. 2:**
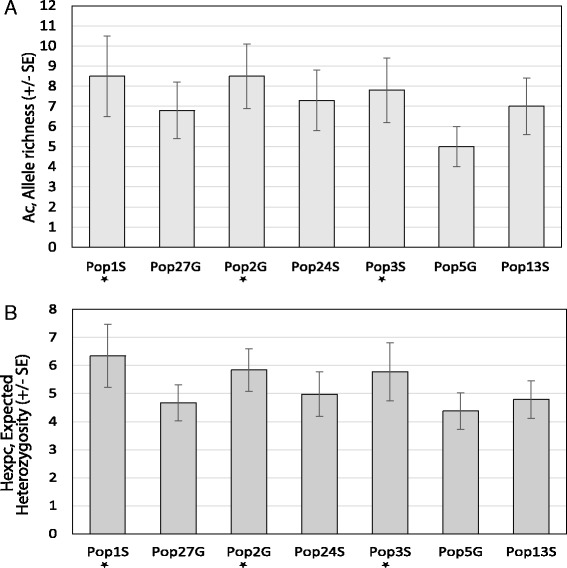
Genetic diversity of the seven *H. contortus* populations based on microsatellite marker genotyping. The corrected mean allelic richness Ac (Panel **a**) and mean expected heterzygosity (H_expc_) (Panel **b**) of the eight microsatellite markers are shown for each population. Error bars indicate the standard error of the mean. H_e_ and allelic richness were calculated per locus for each population with correction for unequal sample size using POPTREE2 and MSA4.05 [31, 32]. The mean H_e_ and allelic richness were compared for statistically significant differences between each pairwise combination of populations using a Wilcox rank sum test. No differences were statistically significant at *P* = 0.05

A panel of eight microsatellites were used to genotype between 14 and 29 individual worms, from each of the seven populations found to contain benzimidazole resistance alleles. Three of these populations were from goats (Pop27G, Pop2G and Pop5G) and four from sheep (Pop1S, Pop24S, Pop3S and Pop13S). *Haemonchus contortus* showed a high level of overall genetic diversity in all populations with a mean number of alleles per locus of 7.250 ± 1.208 (range 2–17) and expected heterozygosity (H_e_) across all loci ranging between 0.646 and 0.707 (Additional file 3: Table S3). Correction of these values for unequal sample size allowed the diversity between populations to be compared; overall corrected allelic richness (A_c_, mean 5.253 ± 0.276) and expected heterozygosity (H_expc_, range 0.642–0.708). There were no statistically significant differences in the overall genetic diversity, as measured by corrected allelic richness or expected heterozygosity, between any of the seven populations (Fig. 2). There were no major departures from linkage equilibrium for any particular combination of loci across all populations indicating that alleles at these loci were randomly associating and not genetically linked (data not shown).

Only 0.003 % of the overall genetic diversity partitioned between populations demonstrating very low genetic sub-structuring of *H. contortus* between the seven locations. This was also reflected by the very low pairwise F_ST_ estimates with only two out of 21 possible pairwise comparisons showing significant values (Table 1). Even in these cases, the F_ST_ values were very low; 0.0175 between Pop2G and Pop24S and 0.0145 between Pop2G and Pop13S. There was also no significant genetic differentiation between the *H. contortus* populations taken from sheep and goats (Table 1). This is consistent with our previous finding in southern India and supports the view that *H. contortus* is freely shared between the two hosts with little or no host species barrier.

**Table 1:** Pairwise F_ST_ values based on genotyping 18 individual worms from each of seven  *H. contortus* populations with eight microsatellite markers

	Pop1S	Pop27G	Pop2G	Pop24S	Pop3S	Pop5G
Pop27G	0.0036					
Pop2G	-0.0089	0.0059				
Pop24S	-0.0057	0.0085	**0.0175**			
Pop3S	-0.0369	0.0041	0.0028	0.0062		
Pop5G	-0.0209	-0.0211	-0.0137	-0.0066	0.0167	
Pop13S	-0.0014	-0.0511	**0.0145**	0.0017	0.0111	-0.0119

Pairwise comparisons with statistically significant genetic differentiation (*P* < 0.001) are highlighted in bold and underlined

### Sequence diversity and phylogenetic relationships of *H. contortus* isotype-1 β-tubulin haplotypes from government farms and pastoral locations

The 328 bp isotype-1 β-tubulin fragment was PCR amplified, cloned and sequenced from pooled DNA from each of the seven parasite populations in which the F200Y (T**A**C) resistance mutation was detected; three populations from government farms (Pop1S, Pop2G and Pop3S) and four populations from rural locations (Pop24S, Pop27G, Pop5G and Pop13S). Following bioinformatic filtering (see Methods), a total of 21 different isotype-1 β-tubulin haplotypes (GenBank accession numbers KR269920–KR269940) were identified in the 89 sequences generated from the seven populations. Six out of the twenty one haplotypes encoded the F200Y(T**A**C) resistance polymorphism. Summary statistics of the isotype-1 β-tubulin fragment diversity are shown in Additional file 4: Table S4.

**Fig. 3:**
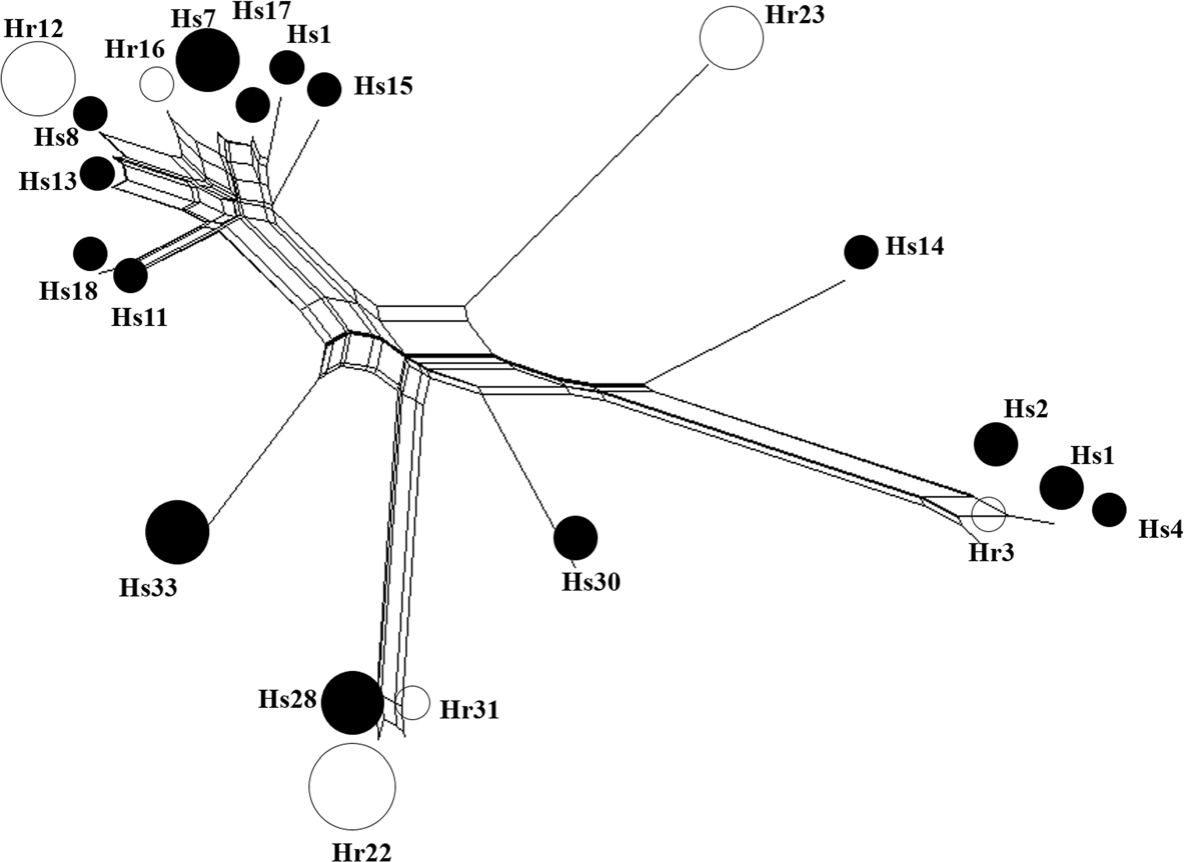
SplitsTree network of 89 *H. contortus* isotype-1 β-tubulin sequences from seven populations (Pop24S, Pop1S, Pop27G, Pop2G, Pop3S, Pop5G and Pop13S) generated with the neighbour-net method of SplitsTree4 [18]. The circles in network represent the different haplotypes and the size of the circles is proportional to the frequency in the overall dataset. The haplotypes containing the different mutations are shaded as follows: susceptible haplotypes containing F200Y (T***T***C)/F167Y (T***T***C)/E198A (G***A***A) are *black*; P200Y resistant haplotypes containing F200Y (T**A**C)/F167Y (T**T**C)/E198A (G***A***A) are *white*

A SplitsTree network was constructed with all twenty one isotype-1 β-tubulin haplotypes to examine their phylogenetic relationships (Fig. 3). The F200Y(T**A**C) resistance polymorphism was present on haplotypes located in four different parts of the network. Each of these resistance haplotypes were more closely related to one or more susceptible haplotypes than to any of the other resistance haplotypes suggesting they were independently derived. A maximum likelihood (ML) tree was also constructed and showed the same phylogenetic relationships (Additional file 5: Figure S2).

### Geographical distribution of F200Y (T**A**C) isotype-1 β-tubulin benzimidazole resistance haplotypes

The three parasite populations from government farms (Pop1S, Pop3S and Pop2G) and one of the parasite populations from a pastoral location (Pop27G) each contained multiple resistance haplotypes. The three remaining parasite populations from rural locations (Pop24S, Pop5G, and Pop13S) contained just a single resistance haplotype (Additional file 6: Figure S1). All four parasite populations from rural locations (Pop24S, Pop27S, Pop5S and Pop13S) showed a high degree of susceptible haplotype diversity whereas the three parasite populations from government farms (Pop1S, Pop3S and Pop2G) did not (Additional file 6: Figure S1).

**Fig. 4:**
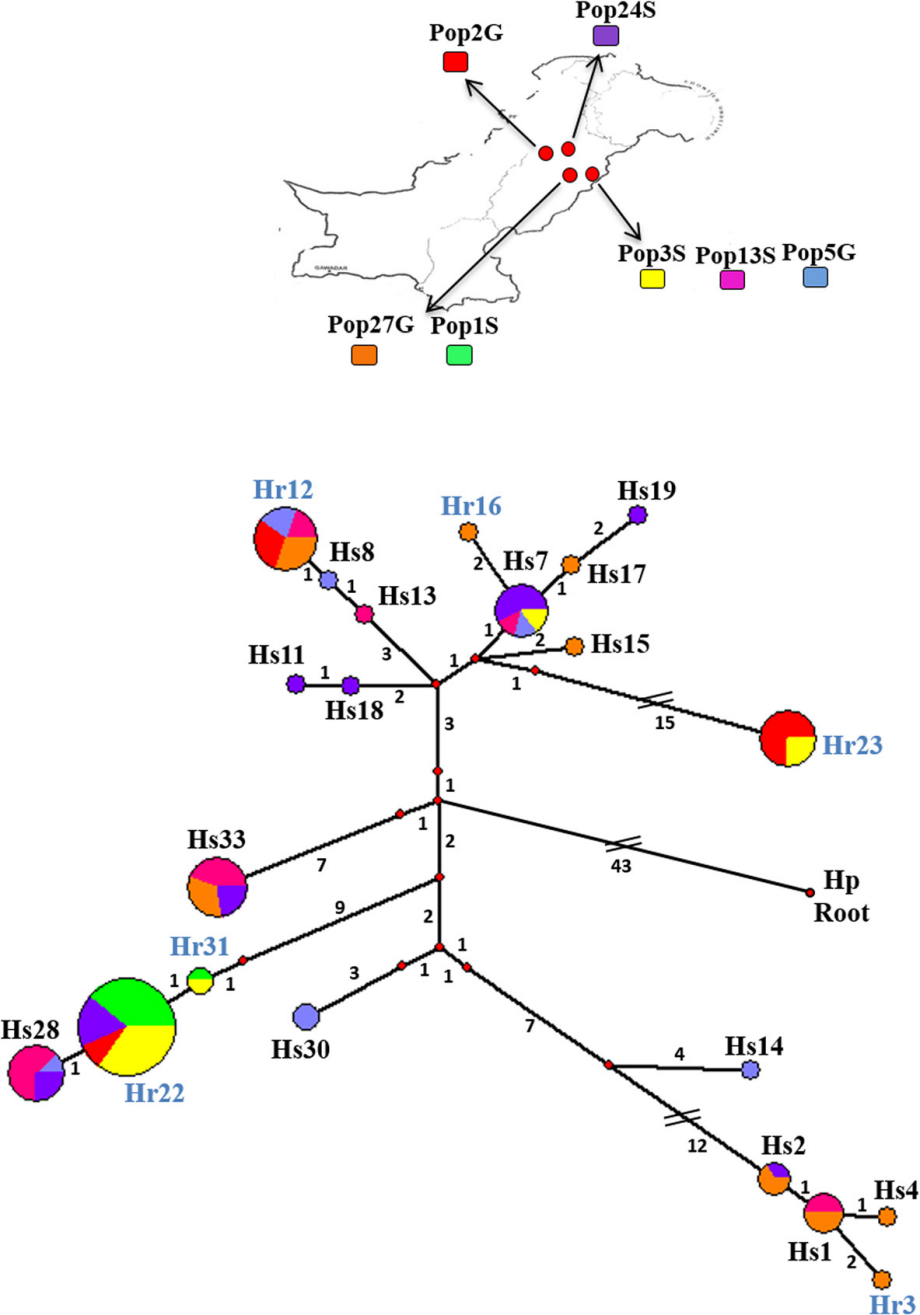
Median joining network of 89 *H. contortus* isotype-1 β-tubulin sequences from seven populations (Pop24S, Pop1S, Pop27G, Pop2G, Pop3S, Pop5G and Pop13S). A full median network containing all possible shortest trees was generated by setting the epsilon parameter equal to the greatest weighted distance (epsilon = 10) using Network 4.6.1 (Fluxus Technology Ltd.). All unnecessary median vectors and links are removed with the MP option [19]. The size of circle representing each haplotype is proportional its frequency in the overall dataset and the colours in the circles reflect the frequency distribution in each geographical location as indicated on the colour key on the inset map. Small red dots represent median vectors. The number of mutations separating adjacent sequence nodes or median vectors is indicated along connecting branches and the length of the lines connecting haplotypes is proportional to the number of nucleotide changes. The most probable ancestral node is determined by rooting the network to a closely related outgroup *H. placei (Hp)* (GenBank accession number KJ598498.1). The text providing the name of each haplotype is colour-coded as follows; susceptible haplotype F200Y (T**T**C)/F167Y (T**T**C)/E198A (G**A**A) is in *black* text; P200Y resistant haplotype F200Y (T**A**C)/F167Y (T**T**C)/E198A (G**A**A) is in *blue* text

A Median-Joining network was constructed to integrate the phylogenetic relationships and frequencies of the different isotype-1 β-tubulin haplotypes with geographical information (Fig. 4). The Median-Joining network was largely congruent with the SplitsTree network and maximum likelihood tree with only minor differences (Fig. 3; Additional file 5: Figure S2). Four of the six resistance haplotypes (Hr22, Hr23, Hr12 and Hr31) were present in multiple parasite populations. Resistance haplotypes Hr23 and Hr31 were present in two different government farms (Pop2G/Pop3S and Pop1S/Pop3S respectively) but were not detected in any of the eighteen pastoral populations. Resistance haplotype Hr22 was present in all three government farms (Pop1S, Pop3S, and Pop2G) as well as in one pastoral population (Pop24S). Resistance haplotype Hr12 was present in just one government farm (Pop2G) and also in several pastoral populations (Pop13S, Pop27G and Pop5G). The two remaining resistance haplotypes (Hr16 and Hr3) were present at low frequency in just a single pastoral population (Pop27G) (Fig. 4).

## Discussion

In most regions of the world examined to date, benzimidazole resistance is at an advanced stage in *H. contortus* with at least one of the known resistance mutations being present at a high frequency in most populations [5–7]. It is difficult to disentangle the population genetics of resistance and draw conclusions about its origins and spread in such regions. In part, this is because of the difficulty of finding natural field populations that have been subject to minimal drug selection. The Punjab region of Pakistan provides an interesting study region in this respect. Small ruminants in pastoral herds in the rural regions of Punjab, Pakistan are rarely treated with effective anthelmintic drugs due to economic and logistical constraints. In contrast, small ruminants on government farms are treated very frequently. This provides the interesting situation in which parasite populations that have been subject to intense drug selection pressure over many years are in close geographic proximity to parasite populations that have been subject to little or no drug selection pressure. We identified three government herds which were founded from locally derived breeding stock over 30 years ago and have been subsequently closed to animal movement. In the case of the Okara (Pop1S) and Jahangirabad (Pop3S) herds, they were derived exclusively from local breeding stock and, although there has been some limited movement of stock between these two farms, they have been otherwise closed to animal movement since their foundation. Further, the sheep on these farms have been treated with benzimidazole drugs approximately every three months over the last 30 years with no history of any other drug class being used. The third government farm in Layyah (Pop2G) was originally established from locally derived goats crossed with angora goats imported from Turkey and has also been closed to animal movement since its foundation. We have compared these government farms with those the surrounding pastoral herds in order to investigate the effects of benzimidazole selection on the genetic diversity of *H. contortus* populations.

As expected from the known patterns of drug use, we found that the F200Y (T**A**C) resistance mutation was present at high frequencies on all three government farms but was absent, or present a very low frequency, in all the pastoral parasite populations sampled. This study thus provides further clear evidence associating the F200Y (T**A**C) mutation with benzimidazole resistance in *H. contortus*; the only three farms out of the 21 examined which had a history of intensive benzimidazole use are also the only ones with a high frequency of this mutation. It is noteworthy that the F167Y (T**A**C) and E198A (G**C**A) mutations were not detected in any of the *H. contortus* populations, including the three government farms subject to intense benzimidazole selection pressure over many years. This is consistent with previous studies from different regions the world that have shown the F200Y (T**A**C) mutation to be essentially ubiquitous in drug selected populations whereas the other two mutations occur more sporadically. For example, in our recent work in southern India the F167Y (T**A**C) mutation was apparently absent and the E198A (G**C**A) mutation, although present at multiple locations, appears to have been derived from a single origin in the region [10]. In the study reported here, the F200Y (T**A**C) mutation was present on multiple haplotypes on each of the government farms; Hr12/Hr22/Hr23 for Layyah (Pop2G), Hr22/Hr31/Hr23 for Jahangirabad (Pop3S) and HR22/Hr31 for Okara (Pop1S). These resistance haplotypes are phylogenetically distant to each other in the haplotype network, being more closely related to one or more susceptible haplotypes than to any of the other resistant haplotypes. This suggests that they were derived from independent origins with the possible exception of haplotypes Hr22 and Hr31 since these are more closely related (Fig. 4). Overall, these new data from Pakistan provide further support for the hypothesis that F200Y (T**A**C) mutations occur commonly and repeatedly whereas the F167Y (T**A**C) and E198A (G**C**A) mutations are much rarer, possibly due to differences in fitness costs between the different mutations.

The occurrence of the F200Y (T**A**C) mutations on multiple divergent haplotypes on each government farm suggests that a soft selective sweep has occurred at the isotype-1 β-tubulin locus in each population (Fig. 4 and Additional file 6: Figure S1). Previous work has demonstrated that soft selective sweeps can occur at the isotype-1 β-tubulin locus in both *H. contortus* and *Teladorsagia circumcincta* [5, 10]. However, these previous studies in the UK and India, were in situations where animal movement was high. Consequently, in those cases the resistance haplotype diversity found at each location could have been due, at least in part, to the migration of resistance haplotypes between farms [5, 10]. However, the government farms in this study have been closed to animal movement for over 30 years and so the soft selective sweeps must have occurred in the absence of significant contemporary migration of resistance alleles between farms.

A variety of studies suggest that laboratory strains of *H. contortus* generally retain high levels of genetic diversity, even if subject to drug selection [34]. However, there is little information on the impact of anthelmintic drug selection on the overall genetic diversity of parasite populations in the field [34]. A comparison of parasite populations on closed government farms in the Punjab, Pakistan, which have a history of frequent benzimidazole drug treatment, with those from neighbouring pastoral herds, that have been subject to little or no drug treatment, provides a valuable opportunity to explore this question. We used a panel of eight previously characterised microsatellite markers to compare the genetic diversity of *H. contortus* populations from three government farms with those from four pastoral herds. There was a high level of allelic polymorphism for all of the microsatellite markers in all seven populations consistent with the high level of genetic diversity expected for this parasite [5, 29, 35]. The parasite populations on the three government farms had a similarly high level of genetic diversity as those from the pastoral herds (Fig. 2). Indeed, there was no statistically significant difference in the overall level of genetic diversity, as measured by microsatellite marker polymorphism, between any of the populations. These results suggest that frequent benzimidazole use over the last 30 years on the three government farms has not resulted in an overall reduction in genetic diversity of the parasite populations despite the loss of virtually all susceptible isotype-1 β- tubulin haplotypes on these three farms. There was also very low population sub-structuring among the seven parasite populations with the pairwise microsatellite Fst values being non-significant between the government farms (Table 1 and Additional file 3: Table S3). This suggests there has been little population bottlenecking or genetic drift, even for those parasite populations under intensive drug selection and closed to contemporary gene flow. This is likely due to the very large effective population sizes of this parasite species maintaining high genetic diversity [34]. One important implication of these results is that genetic changes associated with drug selection, such as loss of polymorphism and linkage disequilibrium, are likely to be largely confined to the regions of the genome surrounding the loci under selection. Although further work, using larger numbers of genome-wide markers, is needed to confirm this hypothesis, it bodes well for the use of genome-wide scans to identify genetic loci underlying anthelmintic drug resistance for this and related parasites.

## Conclusions

The F200Y(T**A**C) benzimidazole resistance mutation was present at very high frequencies in *H. contortus* populations from three government farms, but not from eighteen pastoral herds, in the Punjab region of Pakistan, consistent with known differences in drug treatment history. Multiple F200Y(T**A**C) haplotypes were present in *H. contortus* populations from each of the “closed” government herds suggesting soft selective sweeps have occurred even in the absence of significant contemporary parasite migration. The selection for a high frequency of benzimidazole resistance alleles on government farms did not lead to a loss in overall genetic diversity, based on microsatellite marker genotyping. This observation has a number of important implications. Firstly, the retention of high genetic diversity in drug resistance parasite populations suggests they are likely to retain adaptive capacity to other selective pressures including treatment with other drug classes. Secondly, genetic changes associated with drug selection, such as loss of polymorphism and linkage disequilibrium, may be largely confined to the regions of the genome surrounding the loci under selection.

## Additional files

Additional file 1: Table S1. Allele frequency (%) of non-synonymous SNPs in isotype-1 β-tubulins from 21 *H. contortus* populations across the Punjab province in Pakistan. (DOCX 19 kb) Additional file 2: Table S2. Summary of panel of microsatellites used for population genetics analysis of *H. contortus (DOCX 17 kb)*
Additional file 3: Table S3. Population genetic data for each microsatellite marker from seven populations of *H. contortus* based on the panel of eight microsatellite loci (DOCX 23 kb) Additional file 4: Table S4. Summary statistics of the isotype-1 β-tubulin fragment diversity (XLSX 9 kb) Additional file 5: Figure S2. Maximum likelihood tree of isotype-1 β-tubulin haplotypes cloned and sequenced from the seven *H. contortus* populations. (TIF 575 kb) Additional file 6: Figure S1. Histograms showing frequencies of the resistant and susceptible isotype-1 β-tubulin haplotypes from seven *H. contortus* parasite populations (TIF 831 kb)

## References

[1] Kaplan RM (2004). Drug resistance in nematodes of veterinary importance: a status report. Trends Parasitol.

[2] Gilleard JS (2013). *Haemonchus contortus* as a paradigm and model to study anthelmintic drug resistance. Parasitology.

[3] Waller PJ (1997). Anthelmintic resistance. Vet Parasitol.

[4] McKellar QA, Jackson F (2004). Veterinary anthelmintics: old and new. Trends Parasitol.

[5] Redman E, Whitelaw F, Tait A, Burgess C, Bartley Y, Skuce P, Jackson F, Gilleard J (2015). The emergence of resistance to the benzimidazole anthlemintics in parasitic nematodes of livestock is characterised by multiple independent hard and soft selective sweeps. PLoS Negl Trop Dis.

[6] Chaudhry U, Gilleard J (2015). Molecular analysis of benzimidazole resistance in *Haemonchus contortus* and *Haemonchus placei*.

[7] Brasil BS, Nunes RL, Bastianetto E, Drummond MG, Carvalho DC, Leite RC, Molento MB, Oliveira DA (2012). Genetic diversity patterns of *Haemonchus placei* and Haemonchus *contortus* populations isolated from domestic ruminants in Brazil. Int J Parasitol.

[8] Hoglund J, Gustafsson K, Ljungstrom BL, Engstrom A, Donnan A, Skuce P (2009). Anthelmintic resistance in Swedish sheep flocks based on a comparison of the results from the faecal egg count reduction test and resistant allele frequencies of the beta-tubulin gene. Vet Parasitol.

[9] Silvestre A, Humbert JF (2002). Diversity of benzimidazole-resistance alleles in populations of small ruminant parasites. Int J Parasitol.

[10] Chaudhry U, Redman EM, Raman M, Gilleard JS (2015). Genetic evidence for the spread of a benzimidazole resistance mutation across southern India from a single origin in the parasitic nematode *Haemonchus contortus*. Int J Parasitol.

[11] Barrère V, Alvarez L, Suarez G, Ceballos L, Moreno L, Lanusse C, RK. P. Relationship between increased albendazole systemic exposure and changes in single nucleotide polymorphisms on the β-tubulin isotype 1 encoding gene in *Haemonchus contortus*. Vet Parasitol. 2012;186(3–4):344–9. 10.1016/j.vetpar.2011.11.068 22192770

[12] Chaudhry U, Miller M, Yazwinski T, Kaplan R, Gilleard J. The presence of benzimidazole resistance mutations in *Haemonchus placei* of US cattle. Vet Parasitol. 2014;204(3-4):411-5. 10.1016/j.vetpar.2014.05.025 24916341

[13] Saeed M, Iqbal Z, Jabbar A, Masood S, Babar W, Saddiqi HA, Yaseen M, Sarwar M, Arshad M (2010). Multiple anthelmintic resistance and the possible contributory factors in Beetal goats in an irrigated area (Pakistan). Res Vet Sci.

[14] Hussain T, Periasamy K, Nadeem A, Ellahi Babar M, Pichler R, Diallo A (2014). Sympatric species distribution, genetic diversity and population structure of *Haemonchus* isolates from domestic ruminants in Pakistan. Vet Parasitol.

[15] Chaudhry U, Redman E, Abbas M, Muthusamy R, Ashraf K, Gilleard J (2015). Genetic evidence for hybridisation between *Haemonchus contortus* and *Haemonchus placei* in natural field populations and its implications for interspecies transmission of anthelmintic resistance. Int J Parasitol.

[16] von Samson-Himmelstjerna G, Walsh TK, Donnan AA, Carriere S, Jackson F, Skuce PJ, Rohn K, Wolstenholme AJ (2009). Molecular detection of benzimidazole resistance in *Haemonchus contortus* using real-time PCR and pyrosequencing. Parasitology.

[17] Drummond AJ AB, Buxton S, Cheung M, Cooper A, Duran C, Field M, Heled J, Kearse M, Markowitz S, Moir R, Stones-Havas S, Sturrock S, Thierer T, Wilson A (2012). Geneious v5.6.

[18] Huson DH, Bryant D (2006). Application of phylogenetic networks in evolutionary studies. Mol Biol Evol.

[19] Polzin T, Daneschmand SV (2003). On Steiner trees and minimum spanning trees in hypergraphs. Oper Res Lett.

[20] Tamura K, Peterson D, Peterson N, Stecher G, Nei M, Kumar S (2011). MEGA5:Molecular Evolutionary Genetics Analysis using Maximum Likelihood, Evolutionary Distance, and Maximum Parsimony Methods. Mol Biol Evol.

[21] Posada D (2008). JModelTest: phylogenetic model averaging. Mol Biol Evol.

[22] Redman E, Packard E, Grillo V, Smith J, Jackson F, Gilleard JS (2008). Microsatellite analysis reveals marked genetic differentiation between *Haemonchus contortus* laboratory isolates and provides a rapid system of genetic fingerprinting. Int J Parasitol.

[23] Otsen M, Plas ME, Groeneveld J, Roos MH, Lenstra JA, Hoekstra R (2000). Genetic markers for the parasitic nematode *Haemonchus contortus* based on intron sequences. Exp Parasitol.

[24] Excoffier L, Laval G, Schneider S (2005). Arlequin (version 3.0): an integrated software package for population genetics data analysis. Evol. Bioinformatics Online.

[25] Guo SW, Thompson EA (1992). Performing the exact test of Hardy-Weinberg proportion for multiple alleles. Biometrics.

[26] Rice W (1989). Analyzing tables of statistical tests. Evolution.

[27] Grillo V, Jackson F, Cabaret J, Gilleard JS (2007). Population genetic analysis of the ovine parasitic nematode *Teladorsagia circumcincta* and evidence for a cryptic species. Int J Parasitol.

[28] Hunt PW, Knox MR, Le Jambre LF, McNally J, Anderson LJ (2008). Genetic and phenotypic differences between isolates of *Haemonchus contortus* in Australia. Int J Parasitol.

[29] Silvestre A, Sauve C, Cortet J, Cabaret J (2009). Contrasting genetic structures of two parasitic nematodes, determined on the basis of neutral microsatellite markers and selected anthelmintic resistance markers. Mol Ecol.

[30] Excoffier L, Smouse PE, Quattro JM (1992). Analysis of molecular variance inferred from metric distances among DNA haplotypes: application to human mitochondrial DNA restriction data. Genetics.

[31] Takezaki N, Nei M, Tamura K (2010). POPTREE2: Software for constructing population trees from allele frequency data and computing other population statistics with Windows interface. Mol Biol Evol.

[32] Dieringer D, Schlötterer C (2003). Microsatellite analyser (MSA): a platform independent analysis tool for large microsatellite data sets. Mol. Ecol. Notes.

[33] Wilcoxon F (1945). Individual comparisons by ranking methods. Biometrics.

[34] Gilleard JS, Redman E. Genetic diversity and population structure of *Haemonchus contortus*. Adv Parasitol. 2016;93:31-68 10.1016/bs.apar.2016.02.009 27238002

[35] Blouin MS, Yowell CA, Courtney CH, Dame JB (1995). Host movement and the genetic structure of populations of parasitic nematodes. Genetics.

