# Supplementary material for: Microsatellite marker analysis of Haemonchus contortus populations from Pakistan suggests that frequent benzimidazole drug treatment does not result in a reduction of overall genetic diversity
Source: Parasit Vectors. 2016 Jun 17;9:349. doi: 10.1186/s13071-016-1624-0 (PMC4912736; doi:10.1186/s13071-016-1624-0)
Supplement: Additional file 1: Table S1. — Allele frequency (%) of non-synonymous SNPs in isotype-1 β-tubulins from 21 H. contortus populations across the Punjab province in Pakistan. (DOCX 19 kb) [file 13071_2016_1624_MOESM1_ESM.docx]

**Supplementary Table S1** Allele frequency (%) of SNPs that resulted in an amino acid changes at codons F200Y (T**T**C/T**A**C), F167Y (T**T**C/T**A**C) and E198A (G**A**A/G**C**A) in isotype-1 β-tubulin obtained from twenty one *H. contortus* populations across the Punjab province in Pakistan.

| **Pakistani field populations** | **Host** | **No of worms in each pool** | **P167** | | **P198** | | **P200** | | **Abattoir / Farms location** | **Origin** |
| --- | --- | --- | --- | --- | --- | --- | --- | --- | --- | --- |
|  |  |  | T**T**C | T**A**C | G**A**A | G**C**A | T**T**C | T**A**C |  |  |
| Pop16S | Sheep | 32 | 100 | 0 | 100 | 0 | 100 | 0 | Lahore | Abattoir |
| Pop13S | Sheep | 29 | 100 | 0 | 100 | 0 | 98 | 2 | Lahore | Abattoir |
| Pop24S | Sheep | 27 | 100 | 0 | 100 | 0 | 95 | 5 | Sargodha | Abattoir |
| Pop3S | Sheep | 32 | 100 | 0 | 100 | 0 | 0 | 100 | Jahangirabad | Farm |
| Pop1S | Sheep | 29 | 100 | 0 | 100 | 0 | 21 | 81 | Okara | Farm |
| Pop2G | Goat | 31 | 100 | 0 | 100 | 0 | 0 | 100 | Layyah | Farm |
| Pop5G | Goat | 14 | 100 | 0 | 100 | 0 | 95 | 5 | Lahore | Abattoir |
| Pop7G | Goat | 27 | 100 | 0 | 100 | 0 | 100 | 0 | Lahore | Abattoir |
| Pop6G | Goat | 14 | 100 | 0 | 100 | 0 | 100 | 0 | Lahore | Abattoir |
| Pop4G | Goat | 22 | 100 | 0 | 100 | 0 | 100 | 0 | Lahore | Abattoir |
| Pop8G | Goat | 15 | 100 | 0 | 100 | 0 | 100 | 0 | Lahore | Abattoir |
| Pop10G | Goat | 29 | 100 | 0 | 100 | 0 | 100 | 0 | Lahore | Abattoir |
| Pop27G | Goat | 26 | 100 | 0 | 100 | 0 | 94 | 6 | Okara | Abattoir |
| Pop28G | Goat | 30 | 100 | 0 | 100 | 0 | 100 | 0 | Okara | Abattoir |
| Pop29G | Goat | 32 | 100 | 0 | 100 | 0 | 100 | 0 | Okara | Abattoir |
| Pop31G | Goat | 20 | 100 | 0 | 100 | 0 | 100 | 0 | Sahiwal | Abattoir |
| Pop33G | Goat | 28 | 100 | 0 | 100 | 0 | 100 | 0 | Sahiwal | Abattoir |
| Pop17G | Goat | 32 | 100 | 0 | 100 | 0 | 100 | 0 | Gujranwala | Abattoir |
| Pop19G | Goat | 31 | 100 | 0 | 100 | 0 | 100 | 0 | Gujranwala | Abattoir |
| Pop20G | Goat | 32 | 100 | 0 | 100 | 0 | 100 | 0 | Gujranwala | Abattoir |
| Pop21G | Goat | 26 | 100 | 0 | 100 | 0 | 100 | 0 | Gujranwala | Abattoir |
