# Supplementary material for: Microsatellite marker analysis of Haemonchus contortus populations from Pakistan suggests that frequent benzimidazole drug treatment does not result in a reduction of overall genetic diversity
Source: Parasit Vectors. 2016 Jun 17;9:349. doi: 10.1186/s13071-016-1624-0 (PMC4912736; doi:10.1186/s13071-016-1624-0)
Supplement: Additional file 2: Table S2. — Summary of panel of microsatellites used for population genetics analysis of H. contortus (DOCX 17 kb) [file 13071_2016_1624_MOESM2_ESM.docx]

**Supplementary Table S2** Panel of microsatellites used for population genetics analysis of *H. contortus*

| **Microsatellite** | **Primer sequences (5' → 3')** | **Allele size range (bp)** | **References** |
| --- | --- | --- | --- |
| Hc36 | F: (HEX) gcatagcggcaaggacgtatg  R: catgacgtactctggttgttcg | 138-158 | [Redman, 2015](#_ENREF_21) |
| Hc22193 | F: (NED) ATCCACTTTCACTCCTATATCA  R: GTGTGCGTGTATCTGTTG | 198-225 | [Redman, 2015](#_ENREF_21) |
| Hc3086 | F: (FAM) AAGCCAACAAAAGACAAT  R: CACATATAGAGCACTTCTCTT | 250-400 | [Redman, 2015](#_ENREF_21) |
| Hc53265 | F: (FAM) TGTAGCTGGACTTACTTTAAATA  R: AGAAGTGGAAATGCTAGATG | 155-248 | [Redman, 2015](#_ENREF_21) |
| Hc25 | F: (FAM) acaggagttatgaatttccgg  R: gcttcagtttgaattgcttccc | 170-312 | [Otsen et al., 2000](#_ENREF_18) |
| Hc33 | F: (HEX) atagcggttcggaggggtttc  R: ccccgtcaaataaaaggctaga | 180-240 | [Otsen et al., 2000](#_ENREF_18) |
| Hc8a20 | F: (FAM) caaacttgacccgacctctc  R: agcgcgttgcacaaaacatt | 170-300 | [Redman et al., 2008](#_ENREF_22) |
| Hc3561 | F: (HEX) CCTACATGTCTCCCATATGTC  R: TTAGCGAAGTAATAGCGTGCC | 262-288 | Chaudhry et al., 2015 |
