# Supplementary material for: Microsatellite marker analysis of Haemonchus contortus populations from Pakistan suggests that frequent benzimidazole drug treatment does not result in a reduction of overall genetic diversity
Source: Parasit Vectors. 2016 Jun 17;9:349. doi: 10.1186/s13071-016-1624-0 (PMC4912736; doi:10.1186/s13071-016-1624-0)
Supplement: Additional file 3: Table S3. — Population genetic data for each microsatellite marker from seven populations of H. contortus based on the panel of eight microsatellite loci (DOCX 23 kb) [file 13071_2016_1624_MOESM3_ESM.docx]

**Supplementary Table S3** Population genetic data for each microsatellite marker from seven populations of *H. contortus* based on panel of 8 microsatellite loci

| Population | Hc36  (8^b^) | Hc3086  (29^b^) | Hc8a20  (18^b^) | Hc3561  (8^b^) | Hc25  (22^b^) | Hc33  (5^b^) | Hc22193  (9^b^) | Hc53265  (13^b^) | All loci |
| --- | --- | --- | --- | --- | --- | --- | --- | --- | --- |
| Pop1S (22^a^) |  |  |  |  |  |  |  |  |  |
| N_o_ | 6 | 0 | 1 | 5 | 0 | 6 | 2 | 4 |  |
| H_e_ | 0.7298 | 0.9418 | 0.6922 | 0.5971 | 0.8572 | 0.4657 | 0.2346 | 0.8190 | 0.6672 |
| H_o_ | 0.6250 | 0.7727 | 0.4761 | 0.2941 | 0.6818 | 0.4375 | 0.0500 | 0.5555 | 0.4866 |
| p-value | 0.5446 | 0.0038 | 0.0019 | 0.0014 | 0.2232 | 1.0000 | 0.0006 | 0.0218 |  |
| F_IS_ | 0.1477 | 0.1830 | 0.3174 | 0.5151 | 0.2085 | 0.0625 | 0.7912 | 0.3280 |  |
| A | 5 | 19 | 12 | 4 | 12 | 2 | 4 | 10 | 8.5 |
| Pop27G (23^a^) |  |  |  |  |  |  |  |  |  |
| N_o_ | 2 | 0 | 0 | 1 | 0 | 2 | 0 | 0 |  |
| H_e_ | 0.8083 | 0.9140 | 0.5381 | 0.6955 | 0.7642 | 0.4831 | 0.3410 | 0.8415 | 0.6732 |
| H_o_ | 0.8571 | 0.2608 | 0.5652 | 0.3636 | 0.6956 | 0.3809 | 0.0434 | 0.5652 | 0.4665 |
| p-value | 0.9078 | 0.0000 | 0.4591 | 0.0000 | 0.5219 | 0.3825 | 0.0000 | 0.0083 |  |
| F_IS_ | -0.0619 | 0.7191 | -0.0514 | 0.4830 | 0.0916 | 0.2156 | 0.8750 | 0.3333 |  |
| A | 7 | 15 | 5 | 4 | 9 | 2 | 4 | 8 | 6.7 |
| Pop2G (29^a^) |  |  |  |  |  |  |  |  |  |
| N_o_ | 0 | 0 | 3 | 3 | 0 | 0 |  | 2 |  |
| H_e_ | 0.7701 | 0.9098 | 0.6960 | 0.7632 | 0.8717 | 0.4821 | 0.3543 | 0.8155 | 0.7078 |
| H_o_ | 0.7241 | 0.7586 | 0.5000 | 0.6538 | 0.8620 | 0.5172 | 0.3703 | 0.5925 | 0.6223 |
| p-value | 0.6527 | 0.1003 | 0.0116 | 0.0087 | 0.1167 | 1.0000 | 0.5522 | 0.0191 |  |
| F_IS_ | 0.0607 | 0.1686 | 0.2857 | 0.1457 | 0.0113 | -0.0741 | -0.0462 | 0.2771 |  |
| A | 5 | 17 | 10 | 6 | 13 | 3 | 6 | 8 | 8.5 |
| Pop24S (27^a^) |  |  |  |  |  |  |  |  |  |
| N_o_ | 0 | 4 | 2 | 1 | 0 | 1 | 2 | 1 |  |
| H_e_ | 0.7512 | 0.8956 | 0.5844 | 0.6666 | 0.8008 | 0.5203 | 0.1485 | 0.8039 | 0.6464 |
| H_o_ | 0.9629 | 0.4347 | 0.6000 | 0.1538 | 0.7407 | 0.3846 | 0.1538 | 0.5769 | 0.5009 |
| p-value | 0.1920 | 0.0000 | 0.2187 | 0.0000 | 0.4953 | 0.1658 | 1.0000 | 0.0068 |  |
| F_IS_ | -0.2888 | 0.5201 | -0.0271 | 0.7727 | 0.0763 | 0.2647 | -0.0362 | 0.2863 |  |
| A | 5 | 13 | 5 | 4 | 14 | 4 | 4 | 9 |  |
| Pop3S (29^a^) |  |  |  |  |  |  |  |  |  |
| N_o_ | 0 | 12 | 0 | 1 | 0 | 0 | 1 | 2 |  |
| H_e_ | 0.7761 | 0.8930 | 0.7519 | 0.5214 | 0.8221 | 0.4936 | 0.1389 | 0.8483 | 0.6557 |
| H_o_ | 0.7931 | 0.5294 | 0.5862 | 0.1785 | 0.6896 | 0.4137 | 0.1428 | 0.7407 | 0.5092 |
| p-value | 0.4523 | 0.0000 | 0.0001 | 0.0000 | 0.1086 | 0.4551 | 1.0000 | 0.0288 |  |
| F_IS_ | -0.0222 | 0.4146 | 0.2234 | 0.6616 | 0.1635 | 0.1641 | -0.0285 | 0.1289 |  |
| A | 5 | 11 | 10 | 3 | 14 | 2 | 5 | 12 | 7.7 |
|  |  |  |  |  |  |  |  |  |  |
|  |  |  |  |  |  |  |  |  |  |
|  |  |  |  |  |  |  |  |  |  |
| Pop5G (14^a^) |  |  |  |  |  |  |  |  |  |
| N_o_ | 0 | 6 | 0 | 0 | 2 | 2 | 1 | 2 |  |
| H_e_ | 0.7539 | 0.9500 | 0.6031 | 0.6772 | 0.8804 | 0.5217 | 0.1630 | 0.7463 | 0.6620 |
| H_o_ | 0.7142 | 0.5000 | 0.5714 | 0.5714 | 0.9166 | 0.1666 | 0.1655 | 0.5833 | 0.5238 |
| p-value | 0.1710 | 0.0001 | 0.2861 | 0.0452 | 0.9933 | 0.0268 | 1.0000 | 0.4405 |  |
| F_IS_ | 0.0545 | 0.4909 | 0.0545 | 0.1612 | -0.0431 | 0.6901 | -0.0232 | 0.2261 |  |
| A | 5 | 10 | 5 | 3 | 8 | 2 | 3 | 4 | 5.0 |
| Pop13S (26^a^) |  |  |  |  |  |  |  |  |  |
| N_o_ | 0 | 1 | 1 | 0 | 0 | 1 | 2 | 0 |  |
| H_e_ | 0.7556 | 0.9102 | 0.4914 | 0.7337 | 0.7345 | 0.4909 | 0.1817 | 0.8386 | 0.6421 |
| H_o_ | 0.6538 | 0.5600 | 0.4400 | 0.2307 | 0.7307 | 0.4230 | 0.1923 | 0.8076 | 0.5048 |
| p-value | 0.4205 | 0.0000 | 0.2695 | 0.0000 | 0.3980 | 0.6832 | 1.0000 | 0.8068 |  |
| F_IS_ | 0.1370 | 0.3896 | 0.1066 | 0.6897 | 0.0052 | 0.1406 | -0.0593 | 0.0375 |  |
| A | 6 | 13 | 5 | 6 | 11 | 2 | 3 | 10 | 7.0 |

N_o_, apparent null homozygotes, i.e. number of worms in the population which failed to give an amplification product for a particular marker; H_e_, expected

heterozygosity; H_o_, observed heterozygosity; F_IS_, inbreeding coefficient; P-values indicate a significant deviation from Hardy–Weinberg equilibrium following bonferroni correction; A, number of alleles.

^a^ Total number of individuals genotyped for each population is given in parenthesis under the population name.

^b^ Total number of alleles for each marker across all populations is given in parenthesis below each marker name.

^c^ Mean number of alleles in each population for eight markers.
