# Supplementary figures and images for: Microsatellite marker analysis of Haemonchus contortus populations from Pakistan suggests that frequent benzimidazole drug treatment does not result in a reduction of overall genetic diversity
Source: Parasit Vectors. 2016 Jun 17;9:349. doi: 10.1186/s13071-016-1624-0 (PMC4912736; doi:10.1186/s13071-016-1624-0)

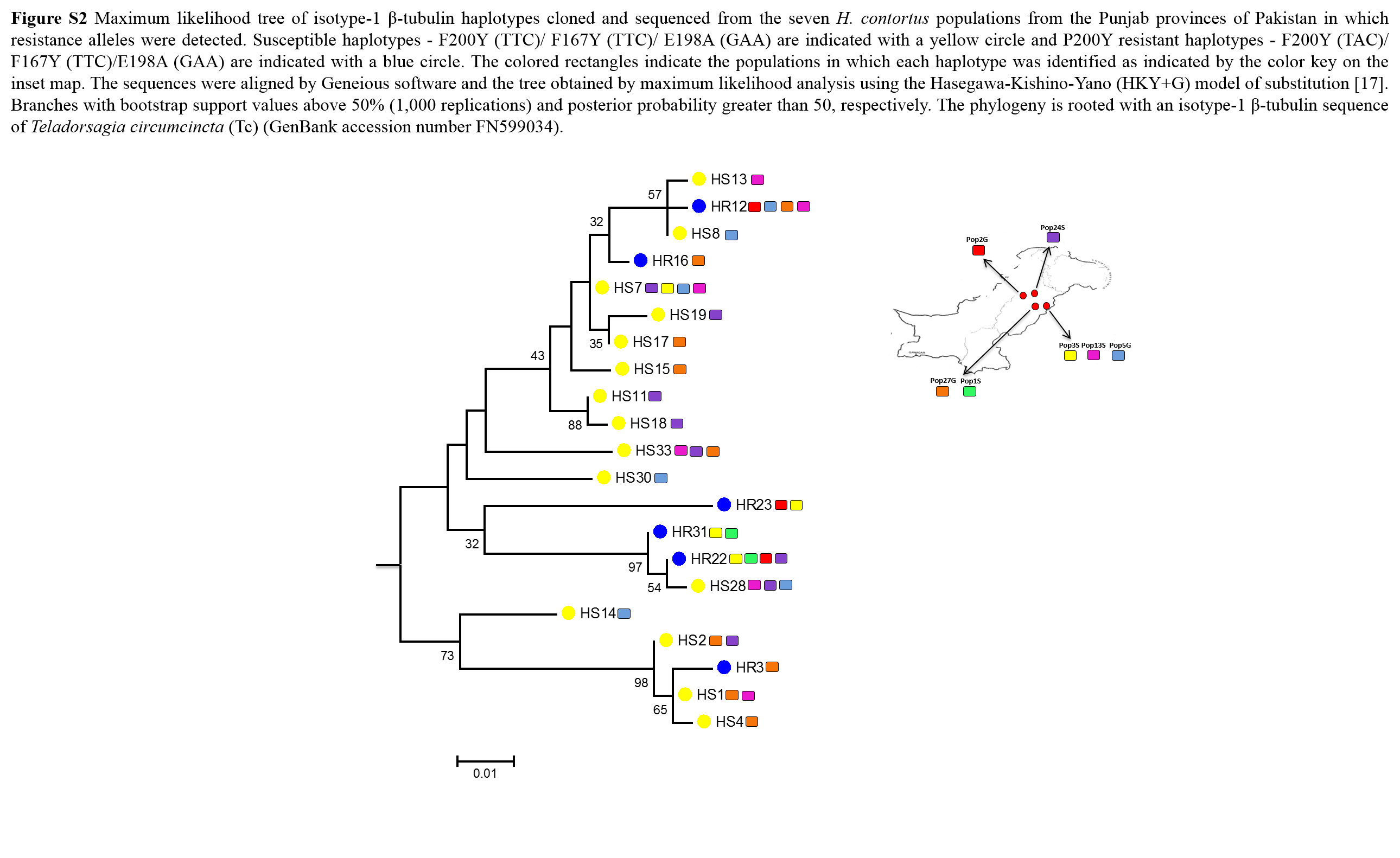

Supplement: Additional file 5: Figure S2. — Maximum likelihood tree of isotype-1 β-tubulin haplotypes cloned and sequenced from the seven H. contortus populations. (TIF 575 kb) [file 13071_2016_1624_MOESM5_ESM.tif]

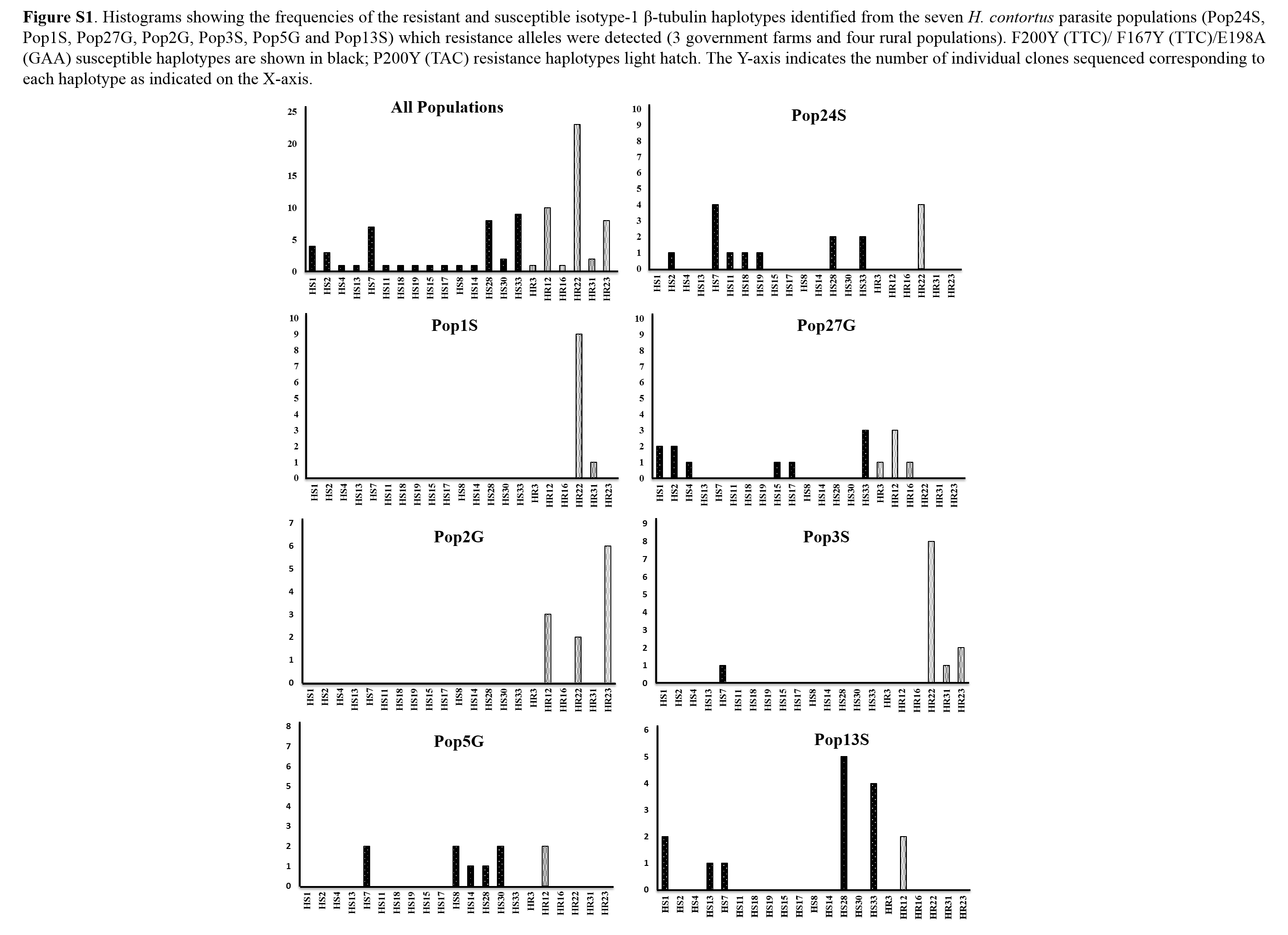

Supplement: Additional file 6: Figure S1. — Histograms showing frequencies of the resistant and susceptible isotype-1 β-tubulin haplotypes from seven H. contortus parasite populations (TIF 831 kb) [file 13071_2016_1624_MOESM6_ESM.tif]
